# Women’s contraceptive profiles in Burundi: Knowledge, attitudes, and interactions with media and health services

**DOI:** 10.1371/journal.pone.0271944

**Published:** 2022-07-27

**Authors:** Kerry L. D. MacQuarrie, Christina Juan, Alison Gemmill

**Affiliations:** 1 The DHS Program, Avenir Health, Glastonbury, Connecticut, United States of America; 2 The DHS Program, ICF, Rockville, Maryland, United States of America; 3 Global Mental Health Division, School of Medicine and Health Sciences, The George Washington University, Washington, DC, United States of America; 4 Johns Hopkins Bloomberg School of Public Health, Baltimore, Maryland, United States of America; FHI360, UNITED STATES

## Abstract

Reproductive health program managers seek information about existing and potential clients’ motivations, behaviors, and barriers to services. Using sequence and cluster analysis of contraceptive calendar data from the 2016–17 Burundi Demographic and Health Survey, we identified discrete clusters characterizing patterns in women’s contraceptive and pregnancy behaviors over the previous 5 years. This study pairs these clusters with data on factors typically targeted in social behavior change interventions: knowledge, attitudes, and women’s interactions with media and health services, to create composite profiles of women in these clusters. Of six clusters, three are characterized by contraceptive use and three are characterized by its absence. Media exposure and attitudes regarding sex preference, wife beating, and self-efficacy largely do not explain cluster membership. Contraceptive knowledge is positively associated with two clusters (Family Builder 1 and Traditional Mother) and negatively associated with a third (Quiet Calendar). Clusters also differ in their members’ fertility desires, contraceptive intentions, and interactions with health services. Two “Family Builder” clusters are both characterized by the presence (but not timing) of multiple pregnancies in their calendar histories, but differ in that women with high contraceptive knowledge, intentions to use contraception, and well-articulated family size ideals are characteristic of one cluster (Family Builder 1), and low contraceptive knowledge, no use of contraception, and vague family size preferences are characteristic of the other (Family Builder 2). These results can guide reproductive health programs as they target social and behavioral change and other interventions to the unique subpopulations they seek to serve.

## Introduction

Researchers have tried to make data on contraception more useful to reproductive health programs by applying market segmentation approaches, with the aim that such programs can better tailor their messages and services to target potential and existing clients. The purpose of market segmentation is to identify distinct subpopulations who have different needs, attitudes, and behaviors around contraception. Segmentation methods group women into different homogeneous profiles (i.e., segments) that characterize their needs and inclinations to use particular services [1]. As human centered design approaches have burgeoned in global health, renewed attention has been given to the segmentation methods these design approaches often incorporate [2–9].

An emerging body of research has used market segmentation approaches to identify targeted groups for family planning interventions. A study in the Philippines, for example, contrasts “young intenders” with older “ready-to-limit” women [10]. In Niger, one study differentiated groups of women who “trust family planning and the health system” from those who “accept limiting” [11], while another study segmented women into “healthy proactives,” “traditional autonomists,” and “modern elites” [12, 13]. The (Re)solve project combines segmentation and behavioral design to, among other goals, develop interventions for adolescent girls based on their trust among peers and (mis)perceptions of pregnancy risk [14]. In other areas of global health, embarrassment and medical concerns versus poor knowledge separated distinct segments of men targeted for voluntary male medical circumcision, while a tuberculosis study segmented treatment seekers along dimensions of perceptions of health systems and social support [15, 16]. These segmentation approaches, which often integrate qualitative data, generally focus on attitudes to form their distinct segments and seldom analyze people’s behaviors.

Demographic and Health Surveys (DHS) contraceptive calendars are a rich source of data on contraceptive behavior and pregnancy experience covering the 5 years preceding the survey [17]. In contrast to this focus on attitudes in most prior segmentation analyses, a recent study has developed methods to identify discrete groups of women based on longitudinal behavioral data from contraceptive calendars in DHS [18]. Similarly, a model has been developed to cluster women into segments based on their contraceptive discontinuation behaviors recorded in DHS calendars [19, 20]. Such behavioral data provide a new and nuanced lens on unique groups of women. However, we do not yet know the attitudinal attributes of the women in these groups.

Attitudes are key determinants of family planning behavior, as are knowledge and interactions with media and health services. Contraceptive knowledge has been positively associated with contraceptive adoption and negatively associated with discontinuation [21, 22]. A broad literature demonstrates that attitudes are associated with the use of modern contraception [21, 23–28]. These attitudes include those toward contraception generally as well as those toward the efficacy and safety of specific methods, self-efficacy, gender, and the role of men. One meta-analysis found that supportive attitudes increase the odds of contraceptive use by an average of 10% to 90%, depending on the specific attitude [29]. As a result, social and behavior change (SBC) interventions seek both to take advantage of supportive attitudes to target services to a clientele inclined to use them, as well as to shift attitudes and social norms to increase demand [30–34]. This same meta-analysis indicated that SBC interventions work to increase modern contraceptive use both directly through their mass media and interpersonal communication activities and indirectly through their effect on attitudes and partner communication [29], with numerous evaluations demonstrating the effectiveness of media exposure and interactions with health providers on contraceptive use [30, 32–34].

In 2018, the government of Burundi established a National Office of the Population to pursue an integrated, cross-sectoral approach to family planning programming [35]. Burundi also focuses on the delivery of community-based health care and scaling up performance-based financing at the community level. The recent Joint Program for Improving the Sexual and Reproductive Health of Adolescents and Youth in Burundi brought together a consortium of donors and implementing partners in common action specifically targeting youth [36]. The 2014 Family Planning Effort (FPE) index revealed high scores in its policy component, driven in part by Burundi’s importation policies, program leadership, and freedom from restrictions on contraceptive advertising [37]. Burundi’s largest improvements were in the services component of the FPE (from 33% in 2009 to 54% in 2014), with top scores in training, logistics, supervision, and staff performance [37]. The country now has the third highest FPE score of the 16 countries assessed in the Francophone/Lusophone Sub-Saharan Africa region [38].

In spite of its strong policy and programmatic focus on family planning, Burundi has the 8th highest total fertility rate among the 86 countries for which DHS has data, at 5.5 children per woman in 2016–17 [39, 40]. Contraceptive prevalence has risen slowly to 18% of all women (29% of currently married women), with injectables and implants being the most commonly used methods. Yet, three in 10 married women have an unmet need for family planning [40]. Scoggins et al estimated government expenditures on family planning to be $976,000 in 2013 [35], levels of domestic funding that have been assessed as needing continued improvement [37]. Together, these indicators would suggest that Burundi’s family planning programming may benefit from the added nuance that behavioral segmentation analysis can provide, so as to better direct its limited resources to address clientele’s specific needs.

This study uses discrete groups of women in Burundi, clustered by their behavioral patterns evident in contraceptive calendar data on their contraceptive and pregnancy experiences over the previous 5 years. We further pair these behavioral clusters with data on knowledge, attitudes, and interactions with media and health services to create rich, composite profiles of women in these clusters. We ask if each of these clusters—distinct in terms of their behavioral characteristics—also differ in terms of the media and health service interactions and attitudinal characteristics associated with them. The composite behavioral-attitudinal profiles are described in detail to offer a nuanced and thorough portrayal of unique clientele groups.

## Methods

### Data

This study uses data from the 2016–17 Burundi DHS survey, which is a nationally representative survey that applied a multistage, clustered sampling process with a response rate of 98.8% among eligible women [40]. Specifically, this study uses contraceptive calendar data from this survey. The contraceptive calendar is completed for all women in the survey and is a retrospective history that records monthly event data on reproductive and contraceptive experiences [17], which we organized into five possible states: (1) no use of contraception; (2) use of a short-term, modern method of contraception; (3) use of a long-acting or permanent method (LAPM) of contraception; (4) use of a traditional method of contraception; and (5) pregnancy, birth, or termination. Short-term, modern methods are pills, injectables, condoms, lactational amenorrhea method, emergency contraception, and the Standard Days Method. LAPMs are intrauterine devices, implants, and sterilization. Traditional methods include periodic abstinence/rhythm, withdrawal, and other traditional or folkloric methods.

### Sequence and cluster analysis

We use calendar sequences that are exactly 59 months long for each woman, with month 1 being the earliest point in the woman’s calendar (approximately 5 years before the interview) and month 59 being the most recent month. Our analytic sample consists of 13,293 women age 15–44 at the start of their calendar sequence (age 20–49 at the time of the survey). Prior to the analysis presented here, we applied sequence and cluster analysis to group the women into 6 distinct contraceptive clusters based solely on patterns of behavior observed in their 59-month calendar sequences. We conducted cluster analysis on the sample of all women aged 15–44 pooled together rather than disaggregated by age group. This full sample analysis allows us to identify broad patterns in the whole population. We can then use age as a covariate to detect the distribution of age groups that fall into each segment rather than imposing, a priori, any sample division based on age or other criteria. Methodological details of the sequencing and clustering procedures used to identify the 6 clusters are documented elsewhere [41].

Briefly, we conducted sequence and cluster analysis in R using the TraMineR and WeightedCluster packages [42–44]. We used a k-medoid (partitioning around medoids, or PAM) clustering algorithm to segment our sample, with Optimal Matching to calculate distances in our dissimilarity matrix and a constant cost matrix to measure pairwise distances between sequences. These parameters and the number of clusters in the final solution were guided by scores on a series of quality metrics [44, 45].

These procedures assign each woman to a single cluster, the one with which her calendar sequence has most in common (in terms of the states she experienced, their sequencing, and timing). Thus, women’s sequences are similar within each cluster and clusters are differentiated from one another by specific defining features. Researchers involved in the study applied descriptive labels to these clusters on the basis of their interpretation of these defining features. The study team reviewed medoid plots, sequence index plots, density plots, and data on mean time, entropy, and turbulence (measures of how static or volatile sequences in a cluster are) to arrive at suitable labels.

### Regression analyses

The clusters are defined by the features of the calendar sequences alone. No other information about the women is used to determine the clusters. Therefore, we estimate separate multivariable logistic regression models to identify women’s attributes associated with membership in each of the identified clusters. Our outcome variable is a dichotomous measure of membership in each cluster. Logistic models offer two advantages over a single multinomial logit model. Separate logistic models allow for a description of all six clusters, which is not possible for the reference cluster in a multinomial model. This was an important goal of this study. Additionally, the results of logistic models are somewhat easier to interpret. A multinomial logit model was tested and produced similar results, confirming that the analysis is not sensitive to the form of the model. For these reasons, we present the preferred logistic models.

For our regression models, we select 7 covariates that describe knowledge and attitudes and 6 covariates that describe media exposure and interactions with the health system. These variables align with conceptual frameworks describing how SBC interventions may influence contraceptive behavior [29]. In particular, the frameworks predict that SBC interventions that engage people through mass media initiatives and interpersonal communication (typically with providers) and influence contraceptive behavior, both directly and indirectly, through increasing knowledge and shifting attitudes and norms [29].

The knowledge and attitudes variables are contraceptive knowledge (categorized as low (0–9 methods), medium (10–11 methods), or high (12–14 methods)), ideal number of children (0, 1–2, 3–4, 5 or more, or non-numeric response), fertility desires (wants a child soon, after 2+ years, unsure of timing, or wants no more), intention to use contraception (currently using, intends to use, does not intend to use), attitudes accepting wife beating in at least one of 5 scenarios, sex preference, and attitudes toward protective self-efficacy. Knowledge and attitudes about contraception and fertility are considered “intermediate outcomes”, influencing contraceptive use and influenced by SBC interventions, in the aforementioned framework [29]. This framework does not consider gender attitudes and norms, but we include them in this same group of variables because of multiple studies showing their association with patterns of contraceptive use [23, 24, 46–49]. We categorize women as having son preference if they preferred more boys than girls, daughter preference if they preferred more girls than boys, and gender balanced/no preference if they expressed no preference or preferred an equal number of girls and boys. Attitudes toward protective self-efficacy is an index summing the number of scenarios, from 0 to 2, in which a respondent believed that a woman is justified in taking action to protect her sexual health. The two scenarios are (1) if a wife is justified in refusing sex with her husband if he has sex with other women, and 2) if a wife is justified in insisting on using a condom if her husband has signs of a sexually transmitted infection.

The media and health service interaction variables are: whether a woman owned a mobile phone or had accessed the internet in the past month, women heard family planning messages on the radio, on TV, in newspapers/magazines, or via mobile phone in the past few months (all coded yes/no), whether a woman visited a health facility or was visited at home by a health worker in the past 12 months and whether family planning was discussed during that visit (no visit; visited, did not discuss family planning; visited and discussed family planning), health insurance coverage (yes/no), and problems seeking medical advice. Problems seeking medical advice are based on women’s responses to whether any of the following presented a “big problem” when she is sick and wants to seek medical advice or treatment: getting permission to go, getting money needed for treatment, distance to the health facility, and not wanting to go alone. Mass media and health service interactions are two categories of SBC interventions considered to affect contraceptive use both directly and indirectly through their influence on knowledge and attitudes [29]. While not explicit in this SBC framework, health insurance and difficulties accessing health services contextualize these intervention variables.

Multivariable models also control for age at the start of the calendar sequence, residence, education, and wealth. These covariates come from cross-sectional data and so we cannot determine causal direction, which is not our intent. It is also not our intent in this study to specify the possible relationships among covariates. While there is some shared structure due to possible associations among these variables, we tested for and found no undue degree of collinearity.

We considered ever had sex as a possible covariate. However, we ultimately excluded it as it perfectly predicted the outcome in most models except the model for the first cluster: Some defining features of the contraceptive clusters, such as pregnancies and births, by definition, coincide with ever having had sex. Therefore, the final set of factors is identical for each of the cluster models.

Regression analyses are conducted in Stata ME 16 and results are presented in odds ratios (ORs). All analyses are weighted to account for sampling probability and nonresponse, and *svyset* commands are used to account for the complex sampling design.

## Results

### Contraceptive clusters

**Fig 1:**
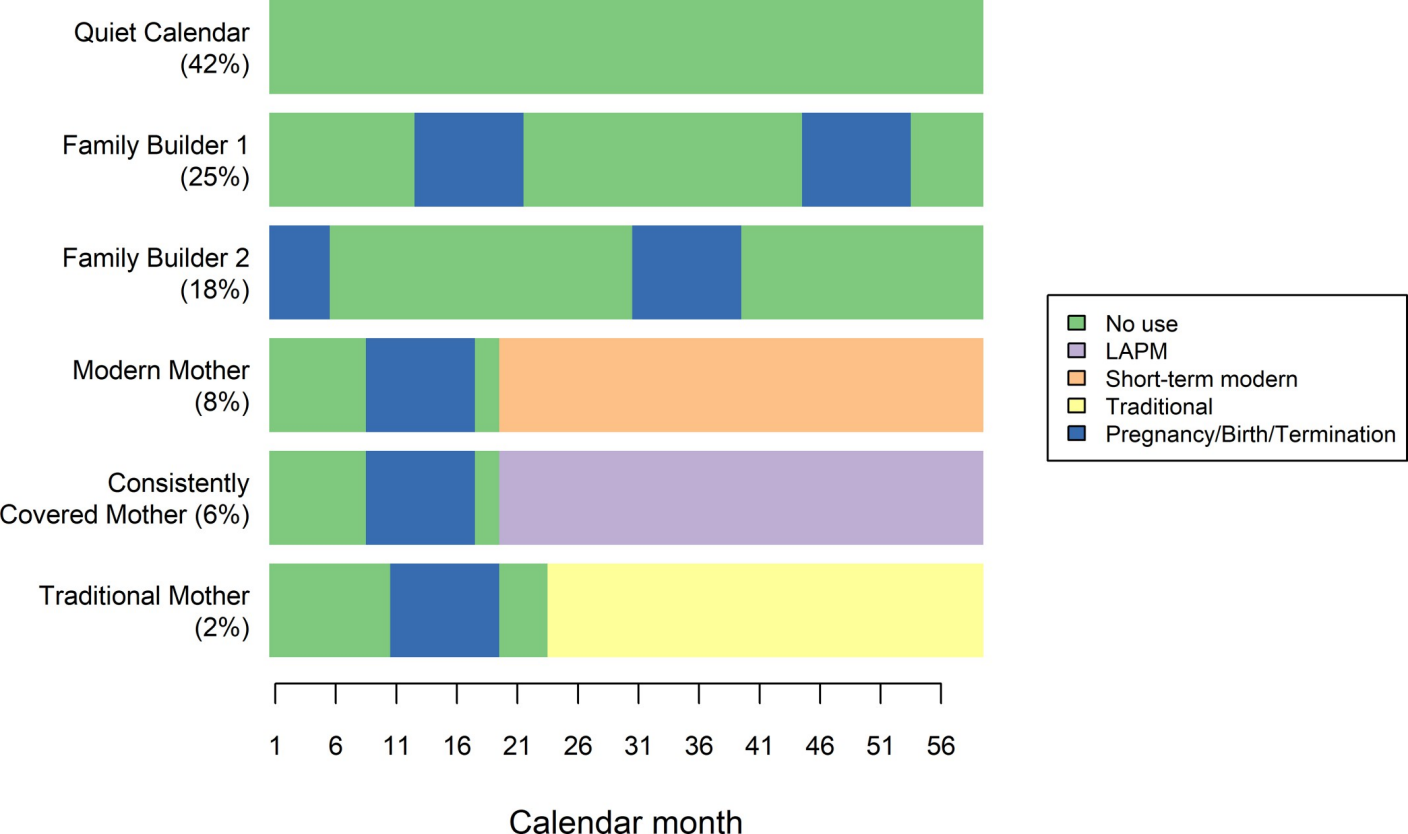
Representative sequence (medoid) and proportion of women in each Burundi contraceptive cluster [41].

We identified 6 distinct clusters from women’s contraceptive calendar sequences in Burundi. These clusters are illustrated by their most representative sequence—the medoid—in Fig 1. They comprise 3 clusters with no discernible contraceptive use: (1) Quiet Calendar (42% of women), characterized by women who did not experience pregnancy or use any methods of contraception; (2) Family Builder 1 (25%) and (3) Family Builder 2 (18%), which are similar in that they are both characterized by women who did not use any method and experienced two pregnancies, but varied in terms of timing during the calendar sequence. The next 3 clusters, all of which are marked by contraceptive use, are: (4) Modern Mother (8%), characterized by women who adopted short-term modern methods toward the end of year 2 after a period of nonuse and a pregnancy; (5) Consistently Covered Mother (6%), characterized by women who adopted LAPMs after a period of nonuse and a pregnancy; and (6) Traditional Mother (2%), characterized by those who adopted traditional methods at the end of year 2 after nonuse and a pregnancy. Women’s calendar sequences that include other experiences, such as intermittent contraceptive use or switching, were not sufficiently prominent in the data to emerge as a separate cluster, because they occurred too infrequently or were insufficiently distinguishable from the six identified clusters. Women with these calendar sequences are grouped in the cluster with the statistically closest medoid sequence. Additional details about the identified clusters are reported elsewhere [41].

### Sample description

Table 1 indicates that the sample is relatively young, with the proportion declining from 24% in the youngest of the five age groups (age 15–19) to 9% in the oldest age group (age 40–44) at the start of the calendar sequence. The sample is largely rural (88%), with most women having either no education (45%) or primary education only (37%). The sample is evenly distributed across household wealth quintiles.

**Table 1:** Analytic sample profile.

	Percent	Weighted n
**Contraceptive Profile Cluster**		
Quiet Calendar	41.5	5,521
Family Builder 1	24.9	3,308
Family Builder 2	18.1	2,400
Modern Mother	7.6	1,007
Consistently Covered Mother	5.6	750
Traditional Mother	2.3	308
**Socioeconomic factors**		
Age at the start of calendar sequence		
15–19	24.2	3,219
20–24	22.6	3,006
25–29	18.3	2,431
30–34	14.6	1,941
35–39	11.5	1,533
40–44	8.8	1,165
Residence		
Urban	12.5	1,666
Rural	87.5	11,627
Highest education level		
No education	44.8	5,955
Primary	36.8	4,896
Secondary or higher	18.4	2,441
Household wealth quintile		
Poorest	20.3	2,696
Poorer	20.2	2,688
Middle	20.1	2,671
Richer	18.9	2,513
Richest	20.5	2,725
**Knowledge and Attitudinal Factors**		
Contraceptive knowledge (# of methods known)		
Low (0–9)	29.2	3,881
Medium (10–11)	26.6	3,538
High (12–14)	44.2	5,874
Ideal number of children		
0	1.5	195
1–2	8.3	1,110
3–4	62.4	8,292
5+	25.7	3,420
Non-numeric response	2.1	276
Sex preference for children		
Balanced or no preference	55.3	7,352
Son preference	30.0	3,993
Daughter preference	14.7	1,948
Attitudes accepting wife beating		
In no scenario	38.6	5,132
In at least one scenario	61.4	8,161
Attitudes accepting self-efficacy (# of scenarios)		
0	14.1	1,880
1	32.5	4,314
2	53.4	7,100
Intention to use contraception in the future		
Using	22.3	2,960
Intends to use	41.4	5,503
Does not intend to use	36.3	4,829
**Interactions with media and health systems**		
Access to internet or mobile phone		
No	73.3	9,748
Yes	26.7	3,545
Heard FP media messages in last few months		
No	68.3	9,082
Yes	31.7	4,211
Visited with health facility or fieldworker in last 12 months		
No visit	18.5	2,455
Visited, did not discuss FP	48.5	6,445
Visited and discussed FP	33.0	4,393
Covered by health insurance		
No	76.8	10,211
Yes	23.2	3,082
Problems seeking medical advice when sick		
None	28.8	3,830
One or more	71.2	9,463
Total	100.0	13,293

For a description of the analytic sample disaggregated by contraceptive cluster, see S1 Table. This table shows some interesting differences in the composition of clusters, which are more rigorously investigated in multivariable analysis. For example, the Quiet Calendar has the highest percentage, 30%, who reported having secondary education or higher, whereas women in the Traditional Mother cluster most commonly have completed primary education (44%) and over one-fifth (22%) have secondary or more education. Women in the remaining clusters typically have no education.

### Composite profiles of each cluster

The regression analyses identify which of the women’s attributes related to knowledge, attitudes, and health system and media exposure are associated with cluster membership. Table 2 presents odds ratios and associated p-values for membership in each of the 6 clusters. Table 3 synthesizes the results from Table 2 by providing summary profiles of each cluster. Combined, the results of these analyses provide composite profiles that richly describe and further differentiate the clusters (as the clusters were originally based only on data from the calendar histories).

**Table 2:** Knowledge, attitudes, and media and health service interactions associated with cluster membership. Odds ratios from logistic regressions.

	Quiet Calendar (42%)		Family Builder 1 (25%)		Family Builder 2 (18%)		Modern Mother (8%)		Consistently Covered Mother (6%)		Traditional Mother (2%)	
	Odds ratio	*p*-value	Odds ratio	*p*-value	Odds ratio	*p*-value	Odds ratio	*p*-value	Odds ratio	*p*-value	Odds ratio	*p*-value
**Socioeconomic factors**												
Age at the start of calendar sequence (ref: 30–34)												
15–19	1.54	<0.001	1.15	0.171	1.00	0.979	0.56	<0.001	0.46	<0.001	0.14	<0.001
20–24	0.60	<0.001	1.42	<0.001	1.27	0.007	0.79	0.106	1.06	0.691	0.43	<0.001
25–29	0.59	<0.001	1.36	<0.001	1.23	0.008	0.85	0.193	1.03	0.852	0.61	0.012
35–39	2.47	<0.001	0.58	<0.001	0.67	<0.001	0.92	0.574	1.14	0.375	0.96	0.843
40–44	10.54	<0.001	0.09	<0.001	0.24	<0.001	0.82	0.314	0.95	0.815	1.77	0.046
Residence (ref: urban)												
Rural	1.05	0.616	1.19	0.064	0.93	0.476	0.61	0.001	1.28	0.131	0.80	0.290
Education (ref: none)												
Primary	1.10	0.130	0.99	0.900	1.04	0.512	0.85	0.119	0.78	0.025	1.24	0.274
Secondary or higher	3.68	<0.001	0.64	<0.001	0.51	<0.001	0.56	<0.001	0.50	0.002	1.16	0.550
Household wealth index (ref: poorest)												
Poorer	0.89	0.154	1.10	0.228	1.06	0.497	0.81	0.085	1.01	0.974	1.05	0.857
Middle	0.91	0.276	1.02	0.845	1.00	0.960	0.76	0.039	1.53	0.005	1.02	0.938
Richer	0.88	0.162	0.98	0.780	1.11	0.233	0.69	0.007	1.32	0.097	1.71	0.030
Richest	0.91	0.422	0.97	0.802	0.95	0.663	1.06	0.752	1.18	0.428	1.56	0.123
**Knowledge and attitudes**												
Contraceptive knowledge (ref: Low (<10 methods))												
Medium (10–11 methods)	0.70	<0.001	1.14	0.051	1.11	0.168	1.22	0.175	1.19	0.265	1.99	0.003
High (12–14 methods)	0.70	<0.001	1.20	0.002	0.95	0.419	1.43	0.006	1.23	0.082	1.83	0.005
Ideal number of children (ref: 1–2)												
0	0.95	0.855	1.15	0.531	1.01	0.961	0.69	0.400	1.09	0.832	1.10	0.886
3–4	0.68	<0.001	1.31	0.005	1.11	0.283	0.88	0.346	1.18	0.323	1.26	0.370
5+	0.46	<0.001	1.79	<0.001	1.22	0.059	0.75	0.069	0.99	0.933	1.05	0.868
Non-numeric response	0.44	<0.001	1.23	0.329	1.83	0.001	1.15	0.710	0.64	0.359	0.72	0.593
Sex preference for children (ref: gender balanced/no preference)												
Son preference	1.15	0.030	0.97	0.622	0.96	0.478	1.08	0.436	0.92	0.423	0.71	0.035
Daughter preference	1.12	0.147	0.96	0.529	1.01	0.930	0.97	0.788	0.77	0.055	1.13	0.503
Attitudes accepting wife beating in at least one scenario												
Yes	1.07	0.161	0.95	0.317	0.89	0.040	1.26	0.007	1.07	0.530	0.88	0.349
Attitudes accepting self-efficacy (# of scenarios, ref: none)												
1	1.04	0.687	1.01	0.913	0.82	0.031	1.46	0.015	1.10	0.548	1.21	0.501
2	1.04	0.684	1.03	0.666	0.82	0.030	1.26	0.113	1.05	0.730	1.44	0.188
Fertility desires (ref: wants within 2 years)												
Wants after 2+ years	0.35	<0.001	3.15	<0.001	1.28	0.009	0.64	0.002	0.54	<0.001	1.11	0.653
Wants, unsure timing	4.21	<0.001	0.29	<0.001	0.30	<0.001	0.35	<0.001	0.21	<0.001	0.45	0.061
Wants no more/sterilized/infecund	0.35	<0.001	2.70	<0.001	1.39	<0.001	0.71	0.015	0.86	0.318	0.70	0.096
Intention to use contraception in the future (ref: does not intend to use)												
Using	0.04	<0.001	1.30	<0.001	0.43	<0.001	29.96	<0.001	21.58	<0.001	32.98	<0.001
Intends to use	0.47	<0.001	1.24	<0.001	1.01	0.888	5.53	<0.001	4.20	<0.001	5.17	<0.001
**Interactions with health services and media**												
Access to mobile phone or internet (ref: no)												
Yes	1.29	0.002	0.86	0.034	0.82	0.012	0.91	0.508	1.55	0.001	1.00	0.997
Heard family planning media messages in last few months (ref: no)												
Yes	1.10	0.160	0.97	0.483	0.90	0.084	1.16	0.094	0.77	0.012	1.28	0.092
Visited with health facility or fieldworker in last 12 months (ref: no visit)												
Visited, did not discuss FP	0.34	<0.001	2.04	<0.001	2.14	<0.001	1.35	0.038	1.26	0.150	1.08	0.725
Visited and discussed family planning	0.18	<0.001	2.92	<0.001	2.41	<0.001	1.37	0.042	1.32	0.115	0.94	0.800
Covered by health insurance (ref: no)												
Yes	0.77	<0.001	1.10	0.101	0.98	0.737	1.22	0.030	1.00	0.965	1.31	0.075
Problems seeking medical advice when sick (ref: none)												
One or more	1.27	<0.001	0.93	0.183	0.94	0.305	0.89	0.229	0.92	0.435	1.06	0.718
Observations	13,293		13,293		13,293		13,293		13,293		13,293	

**Table 3:** Summary profiles of clusters defined by a lack of contraceptive use (panel A) and by contraceptive use (panel B).

	**A. Clusters defined by a lack of contraceptive use**		
	**Quiet Calendar (42%)**	**Family Builder 1 (25%)**	**Family Builder 2 (18%)**
**Socioeconomic factors**	• Adolescents or older women (age 35+) • Educated	• Younger than age 30 • Little education	• Younger than age 35 • Little education
**Knowledge and attitudes**	• Low contraceptive knowledge • No defined plans for children • Smaller ideal family size • Son preference • Do not intend to use contraception	• Medium-High contraceptive knowledge • Larger ideal family size • Defined fertility desires: Desire to avoid or delay pregnancy • Intends to contraception	• Ideal number of children is undefined (e.g. don’t know; up to God) • Somewhat more likely to desire to avoid or delay pregnancy • Mixed gender attitudes: Unaccepting of wife-beating but little support for protective self-efficacy
**Interactions with health services and media**	• Have mobile phones/internet access • Little interaction with health services • No health insurance • Encounter problems seeking medical advice when sick	• No mobile phone/internet access • Recently visited health worker or discussed family planning with a health worker	• No mobile phone/internet access • Recently visited health worker or discussed family planning with a health worker
	**B. Clusters defined by contraceptive use**		
	**Modern Mother (8%)**	**Consistently Covered Mother (6%)**	**Traditional Mother (2%)**
**Socioeconomic factors**	• Not adolescents • Urban • Little education	• Not adolescents • No education	• Older than age 30
**Knowledge and attitudes**	• High contraceptive knowledge • Want a child soon • Using or intend to use contraception • Mixed gender attitudes: Accepting of wife-beating and protective self-efficacy	• Want a child soon • Using or intend to use contraception	• Medium-high contraceptive knowledge • Using or intend to use contraception • No sex preference or preference for gender balance
**Interactions with health services and media**	• Recently visited health worker • Have health insurance	• Have mobile phone/internet access • Have not heard family planning messages in the media	• Interactions with health services and media are not meaningful attributes for this cluster

### Quiet calendar

Overall, Quiet Calendar women tend to be on the very young or older tails of the age spectrum, be educated, and own mobile phones. However, they do not have high contraceptive knowledge nor much interaction with health services. Although they prefer a small family size and prefer sons, they do not have defined plans for having a child (or another child) and do not intend to use contraception.

The unique pattern of association between age and membership in the Quiet Calendar cluster shown in Table 2 is not found in any of the other five clusters. Compared with women in the middle age group (age 30–34), both older women (OR = 2.5–10.5, p<0.001) and adolescent women (OR = 1.5, p<0.001) have higher odds of belonging to the Quiet Calendar cluster, while women in their 20s have lower odds (OR = 0.6, p<0.001).

Women with secondary or higher education have higher odds (OR = 3.7, p<0.001) of being in this cluster than do women without education. Nonetheless, women with medium or high knowledge of contraceptive methods have 30% lower odds (OR = 0.7, p<0.001) of belonging to the Quiet Calendar cluster than do women who have low contraceptive knowledge; this was the only cluster to demonstrate a negative association with contraceptive knowledge.

Multiple attitudinal factors are associated with Quiet Calendar membership. Gender attitudes, however, are not. Women whose ideal family included many children have lower odds of being in the Quiet Calendar cluster than do women who reported 1–2 children as their ideal number of children. Women with a preference for sons have 15% higher odds (p<0.05) of being in the Quiet Calendar cluster than women who preferred gender balance or who had no preference in the sex composition of their children. Sex preference is not a factor in membership in any other cluster model.

When compared with women who wanted a child within 2 years, women who wanted a child but were unsure of the timing have more than four times the odds of being in the Quiet Calendar cluster, while those who wanted to delay children or wanted no more children have 65% lower odds (p<0.001). Women who are using contraception (OR = 0.04, p<0.001) or intended to use contraception (OR = 0.47, p<0.001) have lower odds of being in the Quiet Calendar cluster than women who do not intend to use contraception. Mobile phone and internet access, but not hearing family planning messages in the media, are predictive of cluster membership: Women who had a mobile phone or access to the internet have 29% higher odds (p<0.001) of being in the Quiet Calendar cluster.

Membership in the Quiet Calendar cluster is also associated with little interaction with health services. Having visited a health facility or visited with a health worker in the past 12 months but not discussed family planning (OR = 0.34, p<0.001), having visited and discussed family planning with a health worker (OR = 0.18, p<0.001), and having health insurance (OR = 0.77, p<0.001) are each negatively associated with membership in the Quiet Calendar cluster. Meanwhile, women who encountered one or more problems seeking medical advice when sick have 1.3 times the odds of belonging to the Quiet Calendar cluster when compared with women who did not have such access problems. This factor is not associated with membership in any of the other cluster models.

### Family Builder 1

The Family Builder 1 cluster generally consists of women who are younger than age 30, are not well-educated, and do not own a mobile phone or have internet access, but who have visited recently with a health worker. Cluster membership is also associated with high contraceptive knowledge, having a larger ideal family size, a desire to avoid or delay pregnancy, and current use or intention to use contraception.

Compared with women in the middle age group, younger women have higher odds and older women have lower odds of being in this cluster. Women with secondary or higher education have 36% lower odds of cluster membership than women with no education. Women who had a mobile phone or internet access have 14% lower odds (p<0.001) of being in the Family Builder 1 cluster compared with women who lacked this access, the opposite direction as for the Quiet Calendar.

Women with high contraceptive knowledge have 20% higher odds (p<0.01) of belonging to the Family Builder 1 cluster than women with low contraceptive knowledge. Compared with women who believed that 1–2 was the ideal number of children, women whose ideal was 3–4 children have 31% higher odds, and those whose ideal was 5 or more have 79% higher odds of being in the Family Builder 1 cluster.

Fertility desires have a strong, significant association with cluster membership. Women who wanted no more children have 3.15 times the odds (p<0.001) and those who wanted to delay birth by 2 or more years have 2.7 times the odds (p<0.001) of being in the Family Builder 1 cluster when compared with women who wanted a child soon. Women who were currently using or who intended to use contraception have 24% to 30% higher odds (p<0.001) of being in the Family Builder 1 cluster than do women who did not intend to use contraception.

Women who had visited a health facility or visited with a health worker but did not discuss family planning in the past 12 months have more than two times the odds of membership in the Family Builder 1 compared with women who had no visit. The same women have nearly three times the odds of membership if they had discussed family planning during that visit.

Factors related to residence, wealth, sex preference, gender attitudes, family planning media messages, health insurance, and problems seeking medical advice are not associated with membership in the Family Builder 1 cluster.

### Family Builder 2

The Family Builder 1 and Family Builder 2 clusters resemble each other in terms of the elements in their calendar sequences. Although they share certain behavioral similarities, Table 2 highlights differences between the two clusters. These contrasts and those among the other clusters are further summarized in Table 3.

Like Family Builder 1, the Family Builder 2 cluster consists of younger women who are not well-educated and who lacked mobile phones or internet access. Also as in Family Builder 1, having visited with a health worker but did not discuss family planning in the past 12 months is positively associated with membership in the Family Builder 2 cluster (OR = 2.14, p<0.001), with the magnitude of the association being larger for women who discussed family planning during that visit (OR = 2.41, p<0.001). The Family Builder 1 and 2 clusters are similar in their members’ fertility desires; however, the magnitude of the effect is smaller for Family Builder 2. For the Family Builder 2 cluster, the odds of membership are slightly higher among women who wanted to delay another birth by 2 or more years (OR = 1.28, p<0.001) or have no more children (OR = 1.39, p<0.001); for the Family Builder 1 cluster, the odds of membership are 2.7–3.2 times higher for these women.

The Family Builder 2 cluster differs from the Family Builder 1 cluster in terms of contraceptive knowledge and intentions, ideal family size, and gender attitudes. Women who provided a non-numeric response when asked about their ideal family size have 83% higher odds of being in the Family Builder 2 cluster than women who preferred 1–2 children. In contrast to the model for Family Builder 1, the ideal number of children is not otherwise associated with cluster membership. Unlike Family Builder 1, women who are using contraception have 57% lower odds (p<0.001)—not higher odds—of belonging to the Family Builder 2 cluster than women who did not intend to use contraception. Contraceptive knowledge is not a factor in cluster membership for the Family Builder 2 cluster.

Women belonging to the Family Builder 2 cluster tend to be mixed in their gender attitudes. Those who believed wife beating to be acceptable—the gender *inequitable* perspective—have 11% lower odds (p<0.05) of cluster membership than women who found wife beating unacceptable. However, the other gender equity variable operates in the opposing direction: Women who believed that a woman was justified in protecting her sexual health in at least one scenario—the gender *equitable* perspective—also have 18% lower odds (p<0.05) of belonging to this cluster than women who found protective self-efficacy unacceptable. This finding is robust in that several alternate specifications of these variables and the omission of either of these variables does not change the finding.

Residence, household wealth, sex preference, health insurance, and problems seeking medical advice are not indicative of membership in this cluster, as is also the case with Family Builder 1.

### Modern Mother

The Modern Mother cluster—the most prevalent of the three clusters characterized by contraceptive use—consists of women who have high contraceptive knowledge, wanted to delay/avoid pregnancy, were using or intended to use contraception, and had had a recent visit with a health worker. The women in this cluster tend to be older than age 20, live in urban areas, and lack secondary or higher education.

Several socioeconomic factors are associated with membership in the Modern Mother cluster. Women in the Modern Mother cluster are unlikely to be adolescents. Women age 15–19 at the start of the calendar sequence have 44% lower odds (p<0.001) of cluster membership than women age 30–34. Compared with urban women, women who resided in rural areas also have 39% reduced odds (p = 0.001) of being in this cluster—the only cluster for which residence is a factor. Similar to the Family Builder clusters, women with secondary or higher education also have lower odds of cluster membership. There is some indication that women in the richer wealth quintile have lower odds of belonging to the Modern Mother cluster than do the poorest women, but wealth is otherwise not a factor.

Contraceptive knowledge, fertility desires, and contraceptive intentions are associated with membership in the Modern Mother cluster. Ideal number of children and sex preference are not associated with membership in this cluster, nor in any other cluster characterized by contraceptive use. Women with high contraceptive knowledge have higher odds of membership (OR = 1.43, p<0.01), similar to Family Builder 1 and Traditional Mother clusters. Women who wanted to delay, wanted to avoid, or were unsure of the timing of having another child have lower odds of being in the Modern Mother cluster than do women who wanted a child soon, ranging from 29% lower for women who wanted no more children (OR = 0.71, p<0.05) to 65% lower for women who were unsure (OR = 0.35, p<0.001). The odds of being in the Modern Mother cluster are much higher among women who intended to use (OR = 5.53, p<0.001) or were currently using (OR = 30, p<0.001) contraception when compared with women not using contraception.

Gender attitudes are also mixed in this cluster, but in the opposite direction as in the Family Builder 2 cluster. The odds of Modern Mother membership are higher among those who found wife beating acceptable (OR = 1.26, p<0.01)—the gender *inequitable* perspective—and among those who thought protective self-efficacy was acceptable in one scenario (OR = 1.46, p<0.05)—the gender *equitable* perspective.

As for factors related to media and health service interactions, women who visited with a health worker but did not discuss family planning or those who discussed family planning during a health worker visit in the past 12 months have 35% to 37% higher odds of cluster membership than do those with no health worker visit. When compared with women who lacked health insurance coverage, women with health insurance also have 22% higher odds of being in the Modern Mother cluster—one of two clusters for which health insurance is a factor. Mobile phone or internet access and family planning messages in the media are not associated with membership, nor are problems seeking medical advice when sick.

### Consistently Covered Mother

Women in the Consistently Covered Mother cluster tend to not be adolescents, lack education, and have not heard family planning media messages in the past few months. The cluster consists of women who were more apt to want a child soon, but who were using or intended to use contraception.

Education and hearing family planning media messages are negatively associated with cluster membership, while mobile phone/internet access and contraceptive intentions are positively associated. Like in the Modern Mother cluster, women age 15–19 (and no other age group) have 54% lower odds of cluster membership (p<0.001) than do women age 30–34. Women with either a primary education or a secondary or higher education also have lower odds (22% and 50%, respectively) of being in the Consistently Covered Mother cluster compared with women with no education.

The odds of cluster membership are 23% lower (p<0.05) among women who had heard family planning media messages and 55% higher among those with a mobile phone or internet access (p = 0.001). This is the only cluster for which hearing family planning media messages in the past few months is associated with cluster membership.

Regarding fertility desires, women who wanted to delay or avoid pregnancy have 46% to 79% lower odds of belonging to the Consistently Covered Mother cluster than do women in the reference category. In other words, women who wanted a child soon have greater odds of being in this cluster—a finding similar to that in the Modern Mother model. In spite of these desires, women who intended to use contraception have 4.2 times the odds and women who were currently using contraception have 21.6 times the odds of being in this cluster compared with women who do not intend to use contraception.

In contrast to the other clusters characterized by contraceptive use, women in this cluster do not necessarily have high contraceptive knowledge. Ideal number of children, sex preference, gender attitudes, health insurance, and problems seeking medical advice are not associated with membership.

### Traditional Mother

Contraceptive knowledge and intentions are the only attitudinal factors associated with membership in the Traditional Mother cluster. Women with medium or high contraceptive knowledge have nearly twice the odds (ORs = 1.83–1.99, p<0.01) of being in the Traditional Mother cluster than do women with low knowledge. In a pattern similar to the Modern Mother and Consistently Covered Mother clusters, women who intended to use contraception have more than five times the odds and those who were using contraception have nearly 33 times the odds of being in the Traditional Mother cluster (p<0.001).

Although sex preference is not generally associated with membership in any other cluster model, women who had a preference for sons have 29% lower odds (p<0.05) of being members of the Traditional Mother cluster compared with women who had no preference regarding sex composition. Membership in this cluster is not otherwise associated with attitudes toward ideal family size, gender, or fertility desires. Furthermore, members and non-members do not differ in their interactions with health services and media, including mobile phone/internet access, hearing family planning media messages, health worker visits, health insurance, or problems seeking medical advice.

Age at the start of the calendar sequence shows a positive association with being in the Traditional Mother cluster. Compared with women age 30–34, younger women have lower odds of cluster membership, while those age 40 and older have higher odds of membership.

## Discussion

This study identified 6 clusters of reproductive behavior in Burundi and used data on women’s contraceptive knowledge, attitudes, and interactions with the media and health services to determine associations between these factors and membership in the individual clusters. Three of the clusters are characterized by contraceptive use, and three are characterized by the absence of contraceptive use.

Two of the non-contraception clusters are characterized by family building (i.e., the use of no contraception and the experience of pregnancies, typically two in the preceding 5 years). The Family Building 1 and 2 clusters appear at first glance to be nearly identical, differing only by the timing of pregnancies. Yet, the results of our regression analyses suggest they are, indeed, programmatically meaningful, distinct groups of women.

Although women in both clusters are generally young and have interacted with a health provider recently, in Family Builder 1, women tend to have medium to high contraceptive knowledge and are using or intend to use contraception. They also specify an exact preferred family size, typically desiring moderate to large families (at least 3 children). In contrast, women in the Family Builder 2 cluster are more likely to provide a non-numeric answer about their ideal number of children, suggesting that women who fall into this profile are in a concentrated family building stage but may not believe they can exercise much control over their reproductive lives [50–56]. This contrast comports with other analyses that indicate that women in the Family Builder 1 cluster have more experience with contraception than do women in Family Builder 2, are less likely to experience unmet need, and are more likely to participate in decisions related to contraceptive use [41, 57].

The three clusters characterized by contraceptive use are each characterized by the type of contraception used—short-term, modern methods; LAPMs; and traditional methods. Although women who are members of these clusters may be using contraception, some (including members of the Consistently Covered Mother cluster) may want to have another child soon. This illustrates the dynamic nature of fertility desires over time [58–61], suggesting that, just as women who are not using contraception may need these services soon, women who are contracepting may soon need IUD- or implant-removal services, antenatal care, or other reproductive services in support of their reproductive intentions [62, 63].

Contraceptive knowledge is positively associated with membership in the Family Builder 1 and Traditional Mother clusters and negatively associated with membership in the Quiet Calendar cluster. Surprisingly, it was not associated with membership in either cluster defined by the use of modern methods, suggesting a need for improved counseling on a range of contraceptive methods, including how they work and their efficacy, side effects, and alternatives. That said, fear of side effects remains a pervasive barrier to contraceptive use in Burundi, which reflects legitimate concerns arising from women’s (in)tolerance to uncertain outcomes [64, 65]. Moreover, previous evidence illustrates that just over one-third of contraceptive users in Burundi reported counseling on side effects, emphasizing the need to better train for providers and develop comprehensive, and culturally-informed counseling protocols to address women’s questions and concerns [40, 65].

Gender equitable attitudes are believed to be related to contraceptive use [23, 24, 46]. However, in this study, we found that gender attitudes were seldom associated with membership in clusters based on contraceptive and pregnancy experiences. This is perhaps in line with the finding that women in clusters defined by the use of modern methods were negatively associated with contraceptive knowledge. When they are associated with cluster membership (Family Builder 2 and Modern Mother clusters), attitudes toward violence and women’s protective self-efficacy operated in opposite directions, suggesting that gender attitudes are more complex and nuanced than typically conceptualized.

Mass media initiatives are a common component of SBC interventions and have been shown to increase contraceptive use [29, 66–68]. However, in this study, we found that exposure to family planning messages in the media is largely not associated with cluster membership. Of the analytic sample, 31% of women reported that they were exposed to family planning messages in the last few months. Just over a quarter of the sample reported having access to the internet and a mobile phone. It is possible that the existing types of SBC interventions did not meaningfully reach women because of limited access to the internet and a cell phone. Further research may be needed on the types of SBC interventions that were implemented at this time and whether this population of women should be targeted differently.

We also found that visiting with a health worker and discussing family planning with that health worker is positively associated with membership in the Modern Mother cluster and both Family Builder clusters. For Family Builders, these health worker visits may have been related to maternal health care, given that the women in these clusters have recent pregnancies in their calendar sequences. Their interactions with health services reinforces the importance of counseling about postpartum family planning (and beyond) during antenatal care visits. Recent interaction with health services is negatively associated with membership in the Quiet Calendar cluster—the largest cluster, encompassing more than 4 in 10 women in Burundi. Problems seeking medical care and low contraceptive knowledge are also associated with the Quiet Calendar cluster.

Women in the Quiet Calendar cluster may be worth investigating further as these women tend to be more educated, but also report low contraceptive knowledge and limited access to health services—a somewhat unusual combination of factors and a contrast from women in other clusters. It may be that these women distrust health services, are of a stage of the life course in which they do not perceive health services to be relevant or designed for them, or face other barriers to health services that are unique to them. Therefore, this presents a formidable challenge to reproductive health programs since these women are largely disengaged from health services. Innovative approaches may be required to reach hard-to-reach women with information and connect them to services. Their mobile phone/internet use and media consumption patterns may hint at some viable approaches for this group. Further, our findings suggest Quiet Calendar women may need general health services before contraceptive or pregnancy care is needed.

Our analysis also indicates that the Quiet Calendar is bimodal in terms of its age composition: women who are younger than age 20 and older than age 35 are both more likely to belong to this cluster. Other analysis of the demographic composition of these clusters shows that this cluster is dominated by unmarried women with no children, but also that it includes some older women who are formerly married or have otherwise completed childbearing [41]. This heterogeneity in terms of women’s characteristics nonetheless results in a consistent, homogenous behavioral calendar sequence. This heterogeneous composition also suggests that a single approach to reaching Quiet Calendar women may be insufficient, but that several approaches, each targeting the two different constituent groups of women, may be warranted. For example, ownership of a mobile phone is common in this cluster. However, mobile health apps or outreach by SMS may be effective only for younger, unmarried Quiet Calendar women, but different modalities may be needed to reach older women.

Our analysis was conducted naive to age. That is, we elected not to stratify our analysis by age group. Rather than imposing age a priori as a determining factor, this analytical choice allows us to see patterns in the whole population and observe whether or not age emerges organically as a defining characteristic of behavioral clusters. We find here that there is an age component to the distribution of clusters, and not just for the Quiet Calendar cluster. For example, we find that women in either of the Family Builder clusters are likely to be under age 25, and Traditional Mothers are likely to be composed of women older than age 40. This evidence and evidence from similar analysis in Nepal indicates that there is a life course component to these clusters, which may be a fruitful extension of this research [18]. It may be worthwhile to define life course stage not just as age alone, however, but more holistically as a collection as age, marital status, and parity factors.

### Limitations

This study is novel in its application of sequence and cluster analysis to longitudinal data and comprehensive in adding attitudinal data to create composite profiles. Yet, it has some limitations. First, many of the covariates in these models assess factors at the time of the survey, whereas the clusters are defined by behaviors over the past 5 years. The regression models are not intended to describe causal relationships. Rather, the models are employed to describe the attributes of women constituting each of the identified clusters while controlling for a range of current factors.

Second, this study does not contain any measures of spousal communication or power dynamics, which have been the focus of other studies on women’s empowerment in Burundi [69]. Such factors are often the target of SBC interventions and are seen as one possible pathway that influences modern contraceptive use [23, 27, 46, 70]. However, measures of spousal communication around family planning are not available in the 2016–17 Burundi DHS. We also exclude measures of household or contraceptive decision making. This topic is outside the scope of the current study and is explored elsewhere [57].

Third, we did not consider the programmatic climate that women were exposed to in our analysis. Indeed, recent efforts, such as the Joint Program for Improving the Sexual and Reproductive Health of Adolescents and Youth in Burundi (or “Menyumenyeshe”), aimed to to provide over 1 million young people with quality and comprehensive sexual and reproductive helath care and services during 2016–2020 [36]. Interventions such as these could alter the patterns observed here among those served by the program and are worth considering in future cluster-based research applications.

## Conclusion

This study made use of behaviorally-defined clusters of women—a novel application of segmentation analysis to DHS calendar data. The six discrete clusters of women in Burundi are based on their contraceptive and pregnancy experiences over the past 5 years. This study further identified associated attributes to provide a comprehensive profile of knowledge, attitudes, media exposure, and interaction with health services in these clusters. The results can inform and guide the design of reproductive health programs as they target SBC and other interventions to the unique subpopulations they seek to serve.

## Supporting information

S1 Table Analytic sample profile, by contraceptive cluster. (DOCX)
