# Supplementary material for: Women’s contraceptive profiles in Burundi: Knowledge, attitudes, and interactions with media and health services
Source: PLoS One. 2022 Jul 27;17(7):e0271944. doi: 10.1371/journal.pone.0271944 (PMC9328534; doi:10.1371/journal.pone.0271944)
Supplement: S1 Table — (DOCX) [file pone.0271944.s001.docx]

S1 Table. Analytic sample profile, by contraceptive cluster.

|  | **Quiet Calendar (n=5,521)** | | **Family Builder 1 (n=3,308)** | | **Family Builder 2 (n=2,400)** | | **Modern Mother (n=1,007)** | | **Consistently Covered Mother (n=750)** | | **Traditional Mother (n=308)** | |
| --- | --- | --- | --- | --- | --- | --- | --- | --- | --- | --- | --- | --- |
|  | **Percent** | **Weighted n** | **Percent** | **Weighted n** | **Percent** | **Weighted n** | **Percent** | **Weighted n** | **Percent** | **Weighted n** | **Percent** | **Weighted n** |
| **Socioeconomic factors** |  |  |  |  |  |  |  |  |  |  |  |  |
| Age at the start of calendar sequence |  |  |  |  |  |  |  |  |  |  |  |  |
| 15-19 | 33.4 | 1,845 | 20.6 | 683 | 18.9 | 454 | 14.9 | 150 | 9.2 | 69 | 5.9 | 18 |
| 20-24 | 15.4 | 851 | 29.4 | 971 | 27.5 | 660 | 25.2 | 254 | 27.2 | 204 | 21.5 | 66 |
| 25-29 | 9.7 | 535 | 25.2 | 833 | 24.0 | 576 | 23.2 | 233 | 24.3 | 182 | 23.1 | 71 |
| 30-34 | 10.8 | 599 | 15.8 | 523 | 16.6 | 398 | 20.0 | 202 | 18.9 | 142 | 25.4 | 78 |
| 35-39 | 13.8 | 763 | 8.0 | 264 | 9.9 | 238 | 11.6 | 117 | 14.3 | 108 | 14.0 | 43 |
| 40-44 | 16.8 | 927 | 1.0 | 34 | 3.1 | 75 | 5.1 | 51 | 6.1 | 46 | 10.2 | 31 |
| Residence |  |  |  |  |  |  |  |  |  |  |  |  |
| Urban | 15.8 | 873 | 8.1 | 268 | 8.5 | 203 | 17.7 | 179 | 10.3 | 77 | 21.3 | 66 |
| Rural | 84.2 | 4,647 | 91.9 | 3,039 | 91.5 | 2,197 | 82.3 | 828 | 89.7 | 673 | 78.7 | 242 |
| Highest education level |  |  |  |  |  |  |  |  |  |  |  |  |
| No education | 41.7 | 2,301 | 46.9 | 1,552 | 49.4 | 1,185 | 44.4 | 447 | 49.0 | 367 | 33.5 | 103 |
| Primary | 28.8 | 1,589 | 43.1 | 1,425 | 42.8 | 1,027 | 41.0 | 413 | 40.7 | 305 | 44.4 | 137 |
| Secondary or higher | 29.5 | 1,631 | 10.0 | 330 | 7.9 | 189 | 14.6 | 147 | 10.4 | 78 | 22.1 | 68 |
| Household wealth quintile |  |  |  |  |  |  |  |  |  |  |  |  |
| Poorest | 18.5 | 1,021 | 22.3 | 737 | 23.2 | 558 | 21.3 | 214 | 17.5 | 131 | 11.5 | 35 |
| Poorer | 18.7 | 1,031 | 22.7 | 752 | 22.6 | 542 | 18.5 | 187 | 17.5 | 131 | 14.6 | 45 |
| Middle | 19.6 | 1,082 | 21.1 | 697 | 20.4 | 490 | 17.6 | 177 | 24.2 | 182 | 14.4 | 44 |
| Richer | 18.8 | 1,040 | 18.6 | 614 | 19.9 | 477 | 15.2 | 153 | 20.6 | 155 | 24.3 | 75 |
| Richest | 24.4 | 1,346 | 15.4 | 508 | 13.9 | 334 | 27.5 | 276 | 20.2 | 152 | 35.2 | 108 |
| **Knowledge and Attitudinal Factors** |  |  |  |  |  |  |  |  |  |  |  |  |
| Contraceptive knowledge (# of methods known) |  |  |  |  |  |  |  |  |  |  |  |  |
| Low (0-9) | 37.2 | 2,055 | 24.2 | 799 | 28.5 | 684 | 16.9 | 171 | 18.9 | 141 | 10.1 | 31 |
| Medium (10-11) | 24.4 | 1,348 | 27.7 | 916 | 29.1 | 699 | 26.6 | 268 | 28.4 | 213 | 30.6 | 94 |
| High (12-14) | 38.4 | 2,118 | 48.2 | 1,593 | 42.4 | 1,017 | 56.4 | 568 | 52.7 | 396 | 59.3 | 182 |
| Ideal number of children |  |  |  |  |  |  |  |  |  |  |  |  |
| 0 | 1.6 | 89 | 1.3 | 44 | 1.4 | 34 | 1.2 | 13 | 1.6 | 12 | 1.2 | 4 |
| 1-2 | 9.2 | 508 | 6.7 | 221 | 7.5 | 179 | 10.6 | 106 | 9.3 | 70 | 8.5 | 26 |
| 3-4 | 63 | 3481 | 60.4 | 1998 | 60.1 | 1443 | 66.1 | 665 | 66.8 | 501 | 66.3 | 204 |
| 5+ | 23.5 | 1295 | 30.2 | 1000 | 28.4 | 682 | 20.8 | 210 | 21.5 | 162 | 23.3 | 72 |
| Non-numeric response | 2.7 | 149 | 1.4 | 45 | 2.6 | 62 | 1.3 | 13 | 0.7 | 6 | 0.7 | 2 |
| Sex preference for children |  |  |  |  |  |  |  |  |  |  |  |  |
| Balanced or no preference | 55.9 | 3,087 | 53.8 | 1,779 | 55.8 | 1,338 | 53.8 | 542 | 56.6 | 425 | 58.9 | 181 |
| Son preference | 29.5 | 1,627 | 31.1 | 1,030 | 29.4 | 706 | 32.2 | 324 | 30.9 | 232 | 24.2 | 74 |
| Daughter preference | 14.6 | 807 | 15.1 | 499 | 14.8 | 356 | 14 | 141 | 12.5 | 93 | 17 | 52 |
| Attitudes accepting wife beating |  |  |  |  |  |  |  |  |  |  |  |  |
| In no scenario | 39.3 | 2,171 | 37.9 | 1,255 | 38.8 | 932 | 35.3 | 355 | 37.4 | 280 | 45.1 | 139 |
| In at least one scenario | 60.7 | 3,350 | 62.1 | 2,053 | 61.2 | 1,468 | 64.7 | 651 | 62.6 | 470 | 54.9 | 169 |
| Attitudes accepting self-efficacy (# of scenarios) |  |  |  |  |  |  |  |  |  |  |  |  |
| 0 | 15.6 | 860 | 13.2 | 435 | 16.3 | 390 | 9 | 90 | 10.8 | 81 | 7.5 | 23 |
| 1 | 32.5 | 1,793 | 31.8 | 1,052 | 31.9 | 765 | 35.9 | 361 | 33.6 | 252 | 29.5 | 91 |
| 2 | 51.9 | 2,868 | 55 | 1,821 | 51.9 | 1,245 | 55.2 | 556 | 55.6 | 417 | 63 | 194 |
| Intention to use contraception in the future |  |  |  |  |  |  |  |  |  |  |  |  |
| Using | 3.1 | 171 | 29 | 960 | 15 | 360 | 70.4 | 708 | 69.4 | 520 | 78.6 | 242 |
| Intends to use | 43.7 | 2,413 | 44.4 | 1,467 | 47.6 | 1,143 | 24.8 | 250 | 23.7 | 178 | 16.9 | 52 |
| Does not intend to use | 53.2 | 2,937 | 26.6 | 881 | 37.4 | 898 | 4.8 | 48 | 6.9 | 52 | 4.5 | 14 |
| **Interactions with media and health systems** |  |  |  |  |  |  |  |  |  |  |  |  |
| Access to internet or mobile phone |  |  |  |  |  |  |  |  |  |  |  |  |
| No | 68 | 3,753 | 79.4 | 2,626 | 80.9 | 1,941 | 71.1 | 716 | 70.2 | 527 | 60.3 | 185 |
| Yes | 32 | 1,768 | 20.6 | 682 | 19.1 | 459 | 28.9 | 291 | 29.8 | 223 | 39.7 | 122 |
| Heard FP media messages in last few months |  |  |  |  |  |  |  |  |  |  |  |  |
| No | 68.4 | 3,775 | 69 | 2,283 | 72.1 | 1,731 | 61.3 | 617 | 67.9 | 509 | 53.9 | 166 |
| Yes | 31.6 | 1,745 | 31 | 1,024 | 27.9 | 669 | 38.7 | 390 | 32.1 | 241 | 46.1 | 142 |
| Visited with health facility or fieldworker in last 12 months |  |  |  |  |  |  |  |  |  |  |  |  |
| No visit | 33.5 | 1,847 | 6.4 | 212 | 8 | 193 | 9.6 | 97 | 9.5 | 71 | 11.5 | 35 |
| Visited | 49.5 | 2,734 | 45.1 | 1,493 | 49.2 | 1,181 | 50 | 503 | 49.9 | 374 | 52.4 | 161 |
| Discussed FP at visit | 17 | 940 | 48.5 | 1,603 | 42.8 | 1,027 | 40.4 | 407 | 40.6 | 305 | 36.1 | 111 |
| Covered by health insurance |  |  |  |  |  |  |  |  |  |  |  |  |
| No | 81.2 | 4,480 | 74.8 | 2,473 | 77.6 | 1,863 | 68.3 | 688 | 70.4 | 528 | 58.3 | 179 |
| Yes | 18.8 | 1,041 | 25.2 | 835 | 22.4 | 538 | 31.7 | 319 | 29.6 | 222 | 41.7 | 128 |
| Problems seeking medical advice when sick |  |  |  |  |  |  |  |  |  |  |  |  |
| None | 27.2 | 1,504 | 29 | 958 | 27.7 | 665 | 34.6 | 348 | 32.5 | 244 | 36 | 111 |
| One or more | 72.8 | 4,016 | 71 | 2,350 | 72.3 | 1,736 | 65.4 | 659 | 67.5 | 507 | 64 | 197 |
